# Environmentally Relevant Levels of Depleted Uranium Impacts Dermal Fibroblast Proliferation, Viability, Metabolic Activity, and Scratch Closure

**DOI:** 10.3390/toxics9090211

**Published:** 2021-09-03

**Authors:** Nathan Cruz, Robert Buscaglia, Matthew Salanga, Robert Kellar

**Affiliations:** 1 Department of Biological Sciences, Northern Arizona University, Flagstaff, AZ 86011, USA; ndc59@nau.edu (N.C.); matthew.salanga@nau.edu (M.S.); 2 Department of Mathematics and Statistics, Northern Arizona University, Flagstaff, AZ 86011, USA; robert.buscaglia@nau.edu; 3 Center for Materials Interfaces in Research and Applications (¡MIRA!), Northern Arizona University, Flagstaff, AZ 86011, USA; 4 Department of Mechanical Engineering, Northern Arizona University, Flagstaff, AZ 86011, USA

**Keywords:** uranyl nitrate, skin, Navajo Nation, statistical modeling, wound healing, scratch assay, dermis cell, cellular respiration, growth curve, cytotoxicity

## Abstract

Uranium (U) is a heavy metal used in military and industrial settings, with a large portion being mined from the Southwest region of the United States. Uranium has uses in energy and military weaponry, but the mining process has released U into soil and surface waters that may pose threats to human and environmental health. The majority of literature regarding U’s human health concern focuses on outcomes based on unintentional ingestion or inhalation, and limited data are available about its influence via cutaneous contact. Utilizing skin dermis cells, we evaluated U’s topical chemotoxicity. Employing soluble depleted uranium (DU) in the form of uranyl nitrate (UN), we hypothesized that in vitro exposure of UN will have cytotoxic effects on primary dermal fibroblasts by affecting cell viability and metabolic activity and, further, may delay wound healing aspects via altering cell proliferation and migration. Using environmentally relevant levels of U found in water (0.1 μM to 100 μM [UN]; 23.8–23,800 ppb [U]), we quantified cellular mitosis and migration through growth curves and in vitro scratch assays. Cells were exposed from 24 h to 144 h for a time-course evaluation of UN chemical toxicity. The effects of UN were observed at concentrations above and below the Environmental Protection Agency threshold for safe exposure limits. UN exposure resulted in a dose-dependent decrease in the viable cell count; however, it produced an increase in metabolism when corrected for the viable cells present. Furthermore, cellular proliferation, population doubling, and percent closure was hindered at levels ≥10 μM UN. Therefore, inadvertent exposure may exacerbate pre-existing skin diseases in at-risk demographics, and additionally, it may substantially interfere in cutaneous tissue repair processes.

## 1. Introduction

In the mid 1900s, large-scale U mining for military and industrial applications were conducted on the Colorado Plateau in the Southwestern United States [1,2]. During this time, an estimated 30 million tons of U ore was extracted and transported to mills for processing [3,4,5]. U mills, such as the Shiprock Mill located in New Mexico, worked to extract isotopes ${}^{235}$U and ${}^{238}$U from natural U. The isotope in the highest abundance ${}^{238}$U, known as depleted uranium due to its relatively low radioactivity, is a waste product of the nitric acid enrichment process [6]. This isotope of uranium has been implemented in armor-piercing round ammunition during the Cold War; however, the reduction needed for ${}^{238}$U has resulted in the accumulation of depleted uranium in mill tailings, and it has been claimed that the reduction contributes to a variety of health and environmental consequences [6].

The majority of mines and mills on the Colorado Plateau were decommissioned in 1968; however, their societal impact continues by presenting health risks to nearby residents. Depleted uranium produced by enrichment operations continue to leach from abandoned mills into local water sources and soils [3,5,6,7]. For example, a survey performed across the five agencies of the Navajo Nation detected 75% of the water sources to contain various forms of U, in addition 12.5% of them exceed the United States Environmental Protection Agency maximum contamination limit (USEPA MCL) [5]. Plans and attempts have been made to remove DU from contaminated areas in order to reduce human exposure [8,9,10]. Despite these efforts, large areas across the Colorado Plateau still far exceed the USEPA and Navajo Nation Environmental Protection Agency (NNEPA) U MCL of 30 ppb (30 μg L${}^{-1}$; 0.126 μM), and hence, water and dust from these areas present a significant risk to human health [3,4,11].

Exposure risks include the inhalation of U particulates that accumulate in the bifurcation nodes of the bronchiole tree. Once deposited, U presents its pro-inflammatory activity by inducing oxidative stress in resident macrophages and epithelial cells [6,7,11,12]. Inhalation and ingestion of U has been linked with elevated occurrences of restrictive lung diseases, such as emphysema and pneumoconiosis, present with kidney fibrosis. In many cases, uranium-exposed patients present symptoms, such as spontaneous gas chest, dyspnea, or diabetes [13,14]. The abundance of research on inhalation and ingestion exposure has enabled medical practitioners to accurately understand these diseases and investigate combative treatments for metal poisoning.

Current literature has deficits in investigating the outcomes of U on other tissues, specifically skin vulnerability. A previous study assessing the effects of water exposure to intact skin reported a significant thinning of the integument in UN-exposed rats [15]. Additionally, the same study presented evidence of UNs capability to decrease the dermal cell presence after bathing rats in UN-containing water. Skin exposure has become an increasing concern to residents of the Navajo Nation, especially to those who have pre-existing non-healing chronic illness.

Upwards of 30% of American Indians lack access to maintained public water systems that are infrequently tested and contain metals or other toxicants [5]. Approximately 5.4% of available water used by these communities to bathe and drink contain U at levels exceeding the standards of the Safe Drinking Water Act and may present compounding issues to their health [1,15]. Furthermore, diabetes and diabetic-related illness in American Indians are 60% greater than the general U.S. population [16]. The combination of unregulated water and the elevated risk for diabetes-associated wounds among the Navajo creates the opportunity for U to have its deleterious effects deep into the integument. If U contact occurs with skin at levels found in these waters, it has the potential to cause compromising alterations to resident dermal cells and may lead to an increased risk of skin infection, further complicating healing [2]. For example, studies have demonstrated uranium dissolved in water to be correlated with a decrease in terminal differentiation of keratinocytes, consequently causing deeper penetration into the dermis [17] These same studies exposed biopsies of skin to uranium contaminated water and elucidated even more the danger of topical exposure. When attempting to decontaminate the dermis with ethane hydroxyl biphosphonic acid (EHBP), little success was made and large amounts of DU resided in dermal tissue. Despite supporting evidence of U to penetrate, partition, and induce alterations to intact skin, there is an absence of in vitro research performed to further investigate the nocuous influence of U.

The current study sought to understand how U manifests its harmful effects on factors involved in maintaining healthy dermal tissue. Employing primary dermal fibroblasts and DU in the form of UN, we aimed to elucidate the effects environmentally relevant levels of U (0.1 μM to 100 μM [UN]; 23.8–23,800 ppb [U]) have on dermal cell physiology, proliferation, and percent closure [1,5,17,18,19,20,21,22]. By utilizing primary dermal cells, we were able to model the dermis and U’s effect, all while providing a better understanding of the observations made in previous in vivo studies. We hypothesized that UN exposure would decrease aerobic fibroblast metabolic activity and viable cell count at levels below the EPA MCL. Additionally, we expected human dermal fibroblast proliferation and percent closure to be negatively impacted by UN in relation to time. The results from the study provide foundation work for full thickness wound research and may predict dermal outcomes of future in vivo studies.

## 2. Materials and Methods

### 2.1. Equipment and Software

Cellular metabolism and viability were analyzed using a Synergy HT fluorescent plate reader (Biotek Instrument, Inc; Winooski, VT, USA) with accompanying Gen5 software (Biotek Instrument, Inc; Winoosku, VT, USA; ver: 2.09.1). Incubation settings of Biotek reader were set at 37 °C unless specified otherwise.

### 2.2. Depleted Uranium Solution

UN $\left(UO_{2}{\left(NO_{3}\right)}_{2}\right)$ was purchased from Structure Probe (West Chester, PA, USA; CAS #13520-83-7). Approximately 0.125 g UN was weighed using a Satorius analytical balance (Goettingen, Germany; Model #A200S) and dissolved in 40 mL of fibroblast growth media (FGM; Cell Applications Inc; San Diego, CA, USA; CAT: 116A-500) by vortexing (Scientific Industries, Inc; Bohemia, NY, USA; model: G-560) for 60 s to create a concentrated stock solution. The UN stock solution was vacuum sterilized using a 0.22 μm vacuum filter apparatus (Corning, Inc; Glendale, AZ, USA; CAT: 431118), then vortexed again for 30 s. Molarity of the UN stock solution was calculated based on true weight transferred and experiment dilutions (0.1 μM, 1 μM, 10 μM, and 100 μM) were prepared in FGM.

### 2.3. Human dermal Fibroblast Culture

Human neonatal dermal fibroblasts (hDFn) procured from Cell Applications, Inc. (hDFn; San Diego, CA, USA; cat: 106-5n; lot: 1248) were received as a passage one (P1) cell line. Cells were thawed in a 37 °C heated water bath (Precision Scientific, Inc; Anna Salai, Teynampet; Cat #66643,) then seeded into a Corning T75 tissue culture treated flask (Glendale, AZ, USA; cat: 430725U) containing 10 mL of FGM. Fibroblasts proliferated in a humidified Thermo Fisher incubator (Thermo Fisher Scientific, LLC; Marietta, OH, USA; model: 3110) at 37 °C; 5.0% CO${}_{2}$ / 95% air atmosphere until culture flask were 90% confluent. After 72 h P1 cells were subcultured. Media was removed and replaced with 3–5 mL of TrypLE${}^{\mathrm{TM}}$ Express (Life Technologies Corp; Grand Island, NY, USA; cat: 12605-010) that reacted for three minutes. Afterward, 10 mL of FGM was added to quench trypsinization, and the cell suspension was transferred to a 15 mL centrifuge conical tube (Thermo Fisher Scientific, LLC; Waltham, MA USA; CAT:339651). Cell suspension was pelleted in a centrifuge at 1000 rpm (35×*g*) for 5 min. Supernatant was discarded and cells were resuspended in 2 mL FGM and agitated gently to create a single cell mixture. Total cell yield was performed using Trypan Blue staining (Life Technologies Corp; Grand Island, NY, USA; CAT: 15250-061) with a two-chamber hemocytometer (Hausser Scientific Comp; Horsham, PA, USA). Cell yield count was used to dilute working cell concentration to recommended dilutions for each experiment protocol. For cryopreservation, cells were diluted to a concentration of 5 × 10${}^{5}$ cells mL${}^{-1}$ in FGM (+10% DMSO; Life Technologies Corp; Carlsbad, CA, USA; CAT:TS-20688) and placed in −80 °C freezer (Thermo Fisher Scientific, LLC; Waltham, MA, USA; CAT: EW-44146-01) for 24 h before being stored in a liquid nitrogen locator tank (Thermo Fisher Scientific, LLC; Waltham, MA, USA; CAT: CY50985) for long term storage.

### 2.4. Determining Mitochondrial Activity (PrestoBlue) Assay and Cell Viability (CyQuant Direct)

To determine metabolic activity, uranium-treated cells were incubated in the presence of resazurin, a lipid permeable compound that is reduced by nicotinamide adenine dinucleotide dehydrogenase (NADH) [23]. Quantifying this reaction provided an estimate of a cell’s reduction capability. In its oxidized form, resazurin was lightly fluorescent; however, once reduced the product becomes highly fluorescent (i.e., larger relative fluorescent unit average). This quantifiable characteristic of resazurin permits the differentiation of non-respiring mitochondria from normal metabolically active cells. Non-respiring mitochondria in dead, damaged, or “starved” cells are unable to reduce significant amounts of resazurin, therefore permitting differentiation from normal metabolically active cells [24].

Mitochondrial metabolic activity of hDFn were assayed by quantifying resazurin reduction per the manufacturer’s instructions (Invitrogen, Inc; Wilmington, DE USA; PrestoBlue${}^{\mathrm{TM}}$; CAT: A13261). hDFn cells were seeded in a black clear bottom 96-well tissue culture plate (Corning; cat: 3603) at a density of 1900 cells well${}^{-1}$. Cells were allowed to adhere for 12 h before untreated FGM was removed and replaced once for the duration of the 3-day experiment with FGM containing UN in selective wells. Cells were then exposed for 24, 48, or 72 h before metabolic activity was analyzed by relative fluorescent units (n = 6 for each treatment at every time point). At the time of recording, 0.11 mL of media from control and UN treated cultures were removed leaving 0.09 mL of solution in each well, and 0.01 mL of PrestoBlue${}^{\mathrm{TM}}$ reagent was added to each sample well and incubated at 37 °C for 20 min. Resorufin has a peak absorbance at $\lambda_{ex}$ = 560 nm and a maximum emission $\lambda_{em}$ = 590 nm. A BioTek fluorescence plate reader with filters accommodating 535 nm excitation and 615 nm emission was used to measure fluorescence with “bottom read” function at a GAIN of 35 (n = 6 for each treatment).

Immediately after reading PrestoBlue${}^{\mathrm{TM}}$, fluorescence data from the CyQuant Direct${}^{\mathrm{TM}}$ (Invitrogen, Inc; Wilmington, DE, USA; CAT: C35012) assay were collected to evaluate viable cell count. Viable cell count was assayed using CyQuant Direct${}^{\mathrm{TM}}$ reagent, which is lipid soluble and accumulates in cell nuclei. A secondary reagent, that is not lipid soluble and therefore unable to pass through an intact cell membrane effectively quenches CyQuant Direct${}^{\mathrm{TM}}$ fluorescence marking nuclei with damaged or compromised membrane integrity—differentiating non-viable cells. Therefore, the relative fluorescence unit average is proportional to viable cells present [25]. The fluorescent nucleic acid stain was prepared at a final percent concentration of 0.4% in 11.7 mL of FGM. An included background suppression dye was added at a concentration of 2.1%. In the same 96-well plate, 0.09 mL of CyQuant Direct${}^{\mathrm{TM}}$ solution was added to each sample. Plates were then incubated for 60 min at 37 °C. After 60 min, samples were removed from incubator and plates were read immediately. Excitation energy at $\lambda_{ex}$ = 508 nm was introduced to samples with peak emission $\lambda_{em}$ = 527 nm being recorded and relative fluorescence units quantified with “bottom read” function at a GAIN of 70. Fluorescence data from PrestoBlue${}^{\mathrm{TM}}$ and CyQuant Direct${}^{\mathrm{TM}}$ were normalized to the average relative fluorescence of the control group for each assay. Additionally, metabolic ratio was calculated by taking the quotient of PrestoBlue${}^{\mathrm{TM}}$ to CyQuant Direct${}^{\mathrm{TM}}$ and normalized to control.

### 2.5. Proliferation Curve

Dermal fibroblasts were seeded at a density of 5.0 × 10${}^{3}$ cells per 1.9 cm${}^{2}$ in 24-well culture plates with FGM containing the previously described UN concentrations. Over the 144 h growth curve FGM with or without UN was replaced every 72 h. Six replicate plates were prepared so that cells could be harvested and counted every 24 h for 144 h using a hemocytometer. At time of counting, treatment solutions were removed and 0.126 mL TrypLE${}^{\mathrm{TM}}$ Express was added to each well. After 3 min the reaction was quenched with 0.126 mL FGM and the total cell suspension were transferred to 1.5 mL microcentrifuge vial (Eppendorf; Hamburg, Germany; CAT: 20170038). The cell suspensions were mixed 7 times to break up clumps, and 0.025 mL of cell solution were combined with 0.025 mL of Trypan Blue (Thermo Fisher Scientific, LLC; Grand Island, NY, USA; CAT:15250061) in a 96-well culture plate for each sample to create a final counting solution with a dilution factor of 2. Finally, 0.010 mL of the cell-trypan solution were transferred to a hemocytometer and cells were counted. Every sample count was performed in triplicate then averaged. Averaged cell count were used to calculate total cell population in each well (*n* = 6 for each treatment) using the following formula:

$$cell\;total\;estimate=\left(\frac{x}{y}\times \frac{1}{10,000}\times df\right)\times V_{tot}$$

where, *x* = averaged cell count, *y* = number of hemacytometer squares used for counting, df = dilution factor, and $V_{tot}$ = total cell suspension volume.

### 2.6. Scratch Assay

Uranyl nitrate influence on hDFn cellular migration were determined using previously published scratch assay protocols [26,27]. Human neonatal dermal fibroblasts (5th passage) were grown in a 12-well tissue culture plate for 72 h until 100% confluent in FGM with or without UN. After approximately 72 h of growth, FGM was removed, and the monolayer of cells were scratched using a p200 pipet tip (Andwin Scientific; Schaumburg, IL, USA; CAT: 53508810) that created an average initial scratch width of 1.0 mm ± 0.1 mm. Each well were then rinsed with 0.5 mL Hank’s balanced salt solution (HBSS; Thermo Fisher Scientific, LLC; Grand Island, NY, USA; CAT: 14025092) to remove damaged associated molecular proteins. Afterward, 1 mL of treated or nontreated FGM was added into each well and returned to the incubator. Cells were imaged using Leica DMi1 inverted microscope at 100× magnification. Images were taken every 4 h over a period of 24 h. Cellular migration values were calculated using MatLab (version: R2019a), where images were converted to gray-scale and a filter was applied to accentuate dark and light areas. Dark areas marked the presence of cells and light areas coincided with cell absence. Wound widths were calculated using pixels comparing the ratio of dark and light pixel distributions. Subsequently, data was converted to mm measurements. Measurements of wound width were standardized to initial scratch area to report percent closure (n = 8 for each treatment).

### 2.7. Statistical Analysis

#### 2.7.1. R Version, Models, and Significance Level

All statistical analyses and figures were performed using R (ver: 3.6.2) and RStudio (ver. 1.2.5019). A combination of one-way ANOVA with mixed-effects, two-way ANOVAs with mixed-effects, and linear and polynomial regression models were created to explain variation of the response variables. All power analyses and experiments were evaluated based on a predetermined significance level with an α-level of 0.05 [28]. Diagnostic plots were utilized to ensure modeling assumptions were tenable and can be reviewed in the accompanying R script.

#### 2.7.2. Metabolic Activity and Viable Cell Count Analysis

When investigating metabolic activity and viable cell count, one-way ANOVA models with a random effect were created for each exposure time point. Across the exposure times, the variable repetition (i.e., 1, 2, 3, *…*, k # of assay repetitions) and treatment group were found to play a significant role. A one-way ANOVA with a random effect of repetition allowed the quantification of assay preparation nuances. Altogether, assay repetition accounted for approximately 26.4%–65.4% of the remaining variance. Once the variance of assay preparation was quantified, conclusions were drawn on the effect UN has on metabolic activity and cell viability. Notably, for consistency, all exposure times were fitted with a random-effect of assay repetition, even when the *p*-value was greater than 0.05. Conclusions were unaffected in situations where the random effect was insignificant but remained in the model. Differences between levels of the fixed-effect UN concentrations were determined by pairwise comparisons with *p*-value adjustments done using Tukey’s test if and only if the null was rejected in the ANOVAs (n = 6 for each treatment).

Cellular metabolism models:

$${\hat{y}}_{24}=100-11.5x_{0.1}-21.2x_{1}-32.7x_{10}-39.2x_{100}$$

$${\hat{y}}_{48}=100+4x_{0.1}-8.6x_{1}-15.8x_{10}-28.6x_{100}$$

$${\hat{y}}_{72}=100-9.9x_{0.1}-15x_{1}-19x_{10}-26.8x_{100}$$

Viable cell count models:

$${\hat{y}}_{24}=100-7.8x_{0.1}-18.1x_{1}-25.8x_{10}-29x_{100}$$

$${\hat{y}}_{48}=100-7.3x_{0.1}-20.3x_{1}-25.5x_{10}-33.3x_{100}$$

$${\hat{y}}_{72}=100-12.6x_{0.1}-23x_{1}-27.3x_{10}-36.7x_{100}$$

#### 2.7.3. Metabolic Activity Ratio Analysis

Ratios of metabolic activity per well were calculated by the following formula:

$$Metabolic\;Ratio=\frac{PrestoBlue^{\mathrm{TM}}RFU_{i,j}}{CyQuantDirect^{\mathrm{TM}}RFU_{i,j}}$$

where the numerator is the metabolic activity reading of row *i* in column *j*, and the denominator is the viable cell count in the same row *i* column *j*. All treatment groups were normalized to the average ratio of the control group. Metabolic activity per well was analyzed by a mixed-effects model with the variable plate repetition as a random-effect. Assay repetition accounted for the remaining 50.2–57.8% variance. Differences between levels of the fixed-effect UN concentrations were determined by pairwise comparisons with *p*-value adjustments done using Tukey’s test if and only if the null was rejected in the ANOVAs (n = 6 for each treatment).

Metabolic ratio models:

$${\hat{y}}_{24}=100-4.1x_{0.1}-3.8x_{1}-10.4x_{10}-13.7x_{100}$$

$${\hat{y}}_{48}=100+12.5x_{0.1}+16x_{1}+14.5x_{10}+8.7x_{100}$$

$${\hat{y}}_{72}=100+2.2x_{0.1}+9.7x_{1}+10.6x_{10}+13.7x_{100}$$

#### 2.7.4. Cellular Proliferation Analysis

To evaluate differences in the estimated cell population per day, an additive two-way ANOVA with the factors hour and UN treatment were created. Both variables significantly contributed to explaining the variance of the estimated cell population (*p*-value${}_{tx}$ < 0.0001; *p*-value${}_{hour}$ < 0.0001). Afterward, an interaction between the two variables was investigated and tested against the additive two-way ANOVA model. The evidence supported an interaction between the treatment and hour (*p*-value${}_{tx\times hour}$ < 0.0001), resulting in the following model:

$$\hat{y}=6915-843\alpha_{0.1}-641\alpha_{1}-843\alpha_{10}-1855\alpha_{100}+2867\beta_{48}+$$

$$36263\beta_{72}+73201\beta_{96}+103751\beta_{120}+129751\beta_{144}+4329\alpha_{0.1}\beta_{48}+$$

$$6192\alpha_{1}\beta_{48}-2530\alpha_{10}\beta_{48}-1181\alpha_{100}\beta_{48}-2361\alpha_{0.1}\beta_{72}-$$

$$16900\alpha_{1}\beta_{72}-18385\alpha_{10}\beta_{72}-23276\alpha_{100}\beta_{72}-15011\alpha_{0.1}\beta_{96}+$$

$$472\alpha_{1}\beta_{96}-7759\alpha_{10}\beta_{96}-34408\alpha_{100}\beta_{96}-8823\alpha_{0.1}\beta_{120}+$$

$$6641\alpha_{1}\beta_{120}-3490\alpha_{10}\beta_{120}-59055\alpha_{100}\beta_{120}-30223\alpha_{0.1}\beta_{144}-$$

$$23359\alpha_{0.1}\beta_{144}-44490\alpha_{10}\beta_{144}-45418\alpha_{100}\beta_{144}$$

where α = indicator vector for factor one (tx), and β = indicator vector for factor two (hour). Differences of cell estimates across treatment groups over time were determined using the above model.

Secondary regression modeling began by using backward model selection testing the additive effect of the variable hour, time passed since inoculation, and UN. The additive effect of both variables significantly explained a proportion of the variance in the response cell estimate (i.e., number of cells in the well; *p*-value${}_{hour}$ < 0.0001; *p*-value${}_{UN}$ < 0.0001). The resulting model, however, violated the assumptions of Linear Regression Theory (LRT). To address the non-normality of residuals, a natural log transformation was applied to the response and the variable hour. The finalized model assimilated to all assumptions of LRT. Using Akaike’s Information Criterion (AIC) as a measurement of fit, the model that included Ln(cell estimate) and Ln(hour) was favored by reducing the AIC value a substantial amount (AIC${}_{non-log}$ = 3989.06; AIC${}_{Ln}$ = 192.48). Conclusions were drawn using the following model, where both the cell estimate and hour were natural log transformed:

Cell proliferation model:

$$Ln\left(cell\;estimate\right)=2.79-0.08z_{0.01}-0.07z_{1}-0.27z_{10}-0.55z_{100}+1.8Ln\left(x_{hour}\right)$$

#### 2.7.5. Population Doubling Analysis

Population doublings (PD) were calculated using the following formula [29]:

$$PD=log_{10}{\left(\frac{n}{n_{0}}\right)}^{3.32}$$

where *n* = cell total estimate, and $n_{0}$ = number of cells seeded. Similar to the cell estimate, differences of PD at each time point were evaluated using an additive and interaction two-way ANOVA model using the variables UN treatment and hour. From the additive model, the evidence supported treatment and hour to significantly contribute to decreasing variance (*p*-value${}_{tx}$ < 0.0001; *p*-value${}_{hour}$ < 0.0001). However, when compared to an interaction two-way ANOVA model, the product coefficients returned as significant (*p*-value${}_{tx\times hour}$ = 0.000126). Differences of PD across treatment group and time were drawn from the following model:

$$\hat{y}=0.40-0.25\alpha_{0.1}-0.11\alpha_{1}-0.17\alpha_{10}-0.41\alpha_{100}+0.41\beta_{48}+$$

$$2.7\beta_{72}+3.6\beta_{96}+4.0\beta_{120}+4.3\beta_{144}+0.68\alpha_{0.1}\beta_{48}+$$

$$0.76\alpha_{1}\beta_{48}-0.31\alpha_{10}\beta_{48}-0.01\alpha_{100}\beta_{48}-0.16\alpha_{0.1}\beta_{72}-$$

$$0.67\alpha_{1}\beta_{72}-0.67\alpha_{10}\beta_{72}-0.85\alpha_{100}\beta_{72}-0.11\alpha_{0.1}\beta_{96}+$$

$$0.06\alpha_{1}\beta_{96}-0.0003\alpha_{10}\beta_{96}-0.48\alpha_{100}\beta_{96}+0.12\alpha_{0.1}\beta_{120}+$$

$$0.16\alpha_{1}\beta_{120}+0.11\alpha_{10}\beta_{120}-0.72\alpha_{100}\beta_{120}-0.11\alpha_{0.1}\beta_{144}-$$

$$0.16\alpha_{0.1}\beta_{144}-0.4\alpha_{10}\beta_{144}-0.2\alpha_{100}\beta_{144}$$

where α = indicator vector for factor one (tx), and β = indicator vector for factor two (hour).

An additive and interaction regression model of UN and hour were compared to adequately explain variation of PD. The interactive term between hour and UN was determined to be insignificant in reducing the mean squared error (*p*-value = 0.8297); therefore, an interaction model was not implemented in the regression analysis. Further transformations were applied to ensure the homoscedasticity and normality of residuals were tenable. The favored model applied a significant 4th-order polynomial (*p*-value < 0.0001) to the variable hour and substantially reduced the AIC coefficient compared to a first-degree, quadratic, and cubic transformations (AIC${}_{simple}$ = 327.61; AIC${}_{quadratic}$ = 276.16, AIC${}_{cubic}$ = 240.87, AIC${}_{quartic}$ = 223.99). A fifth-order polynomial was determined to insignificantly decrease the error (*p*-value = 0.8696) and thus presented evidence for the finalized quartic model. Finally, the resulting 4th-order polynomial function increased the proportion of variance explained to greater than 90% (R${}_{adj}^{2}$ = 0.9314). Differences in the predicted average PD across UN concentrations over time were drawn from the developed model:

PD model:

$$\hat{y}=2.93+20.69x-3.99x^{2}-2.93x^{3}+1.92x^{4}-0.12z_{0.1}-0.09z_{1}-0.38z_{10}-0.8z_{100}$$

#### 2.7.6. hDFn Percent Closure Analysis

Individual differences of the UN treatment at discrete time points were evaluated using an additive two-way ANOVA model with a mixed-effect plate preparation. The variables, UN treatment and hour, were both found to be significant in the additive model (*p*-value${}_{tx}$ < 0.0001; *p*-value${}_{hour}$ < 0.0001). Interestingly, an interaction term between the two variables did not significantly decrease the variance (*p*-value${}_{tx\times hour}$ = 0.09112). Therefore, differences across all time points are equal and were drawn from the following function:

$$\hat{y}=25+2.7\alpha_{0.1}-11.5\alpha_{1}-13.1\alpha_{10}-10.2\alpha_{100}+27.6\beta_{8}+48.4\beta_{12}+$$

$$63.1\beta_{16}+69.5\beta_{20}+74.8\beta_{24}$$

where α = indicator vector for factor one (tx), and β = indicator vector for factor two (hour). Plate preparation accounted for 35.4% of any remaining variance not explained by the additive main-effects.

Similar to Section 2.7.4, regression modeling of the cellular percent closure was conducted through backward variable selection and included hours post-scratch and UN as fixed-effects. The additive effect of both variables was concluded to be significant (*p*-value${}_{hours\;post-scratch}$ < 0.0001; *p*-value${}_{UN}$ < 0.0001). Afterward, an interaction effect of the two variables was observed to significantly explain percent closure variation (*p*-value = 0.04435). However, the interaction model violated the assumption of LRT, specifically the assumption of homoscedasticity. To address the violation, a natural log transformation was applied to the time variable, and in doing so significantly decreased the mean square error (*p*-value = 0.00727). Afterward, the transformed model ensured all model assumptions were tenable. The natural log transformed model was also favored against the non-log transformed interaction model by lowering the AIC value by 83.5 units (AIC${}_{simple}$ = 1635.23; AIC${}_{final}$ = 1551.73). Finally, the concluding model substantially increased the percent of variation explained by the response variable (R${}_{adj-simple}^{2}$ = 0.7987; R${}_{adj-final}^{2}$ = 0.8663). Contrasts of percent closure between the levels of UN was conducted every 4 h along the Ln(hours post-scratch) domain using the favored model.

Cell percent closure model: -4.6cm0cm

$$percent\;closure=-32+13z_{0.1}-8.1z_{1}-28.9z_{10}-27.5z_{100}+42.1Ln\left(x_{hour}\right)-5.5Ln\left(x_{hour}\right)z_{0.1}$$

$$-1.4Ln\left(x_{hour}\right)z_{1}+6.6Ln\left(x_{hour}\right)z_{10}+7.3Ln\left(x_{hour}\right)z_{100}$$

## 3. Results

### 3.1. UN Significantly Impairs Metabolic Activity and Viable Count of Dermal Cells at Levels Below EPA MCL

**Figure 1:**
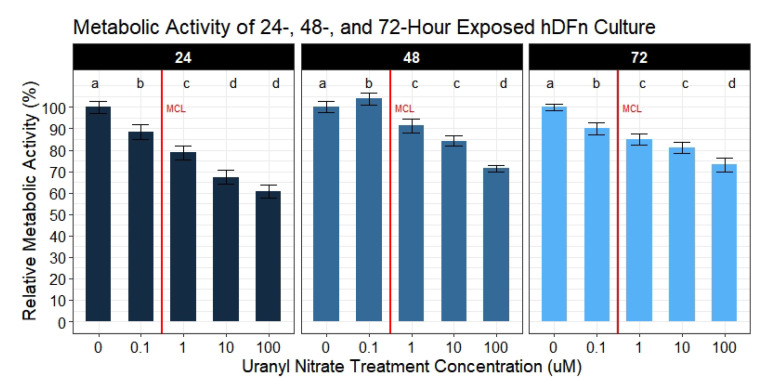
Metabolic activity results of UN exposured hDFn. UN decreases PrestoBlue${}^{textTM}$ readings in cultured human dermal cells at levels below and above the EPA MCL. Bar charts are faceted by exposure time representing the mean metabolic activity percentage ±StdErr of each treatment group (n = 6 done in sextuplicate). All UN concentrations are in μM with -0- representing control group. Statistically significant differences between groups are illustrated by compact letter display with different letters at the top representing a balanced Tukey pairwise *t*-tests where *p*-value ≤ 0.05. Group means followed by a common letter were not different at 5% level of significance. Vertical red line illustrates the EPA MCL threshold for uranium (0.126 μM).

The cellular metabolism was diminished in dermal fibroblasts proportionally to increasing levels of UN, as indicated by a significant decline in resazurin reduction (Figure 1). After 24 h of exposure, a statistically significant decrease in metabolic activity was measured in cells exposed to 0.1–100 μM UN compared to control. A dose-dependent effect was absent in cells treated with concentrations greater than or equal to 10 μM. After 48 h of exposure, the effective dose (ED), which is the concentration of UN to cause a significant decrease in metabolic activity, was raised to 1 μM (Figure 1). Notably, this concentration is above the EPA MCL and is already deemed not safe for human exposure; nonetheless, this level is found in abundance in the United States Southwest. Interestingly, hDFn cells after 48 h of exposure to UN caused a significant 4% (95% CI, −1–8.8) average increase in relative metabolic activity at the lowest dose; however, this effect was short lived. Accompanying this result, dermal cells cultured with higher concentrations of UN were observed to have a smaller range of effects on metabolic activity at 48 h. UN above the MCL decreased the relative cell metabolic activity on average between 8.6% and 28.6% (95% CI, 3.8–33.4), whereas after only 24 h of exposure, the range of effects was greater, falling between 11.5% and 39.2% (95% CI, 6.6–44.2). After a total of 72 h of exposure, the ED on cells was observed again to be below the EPA MCL of U (Figure 1). Levels below the EPA MCL resulted in an average of 9.9% (95% CI, 5.8–13.9) less metabolic activity relative to control. Finally, at 72 h, the decrease in the cellular metabolism had a narrow range of 9.9%–26.8% (95% CI, 5.8–30.8) across all treatment concentrations compared to other exposure timepoints.

**Figure 2:**
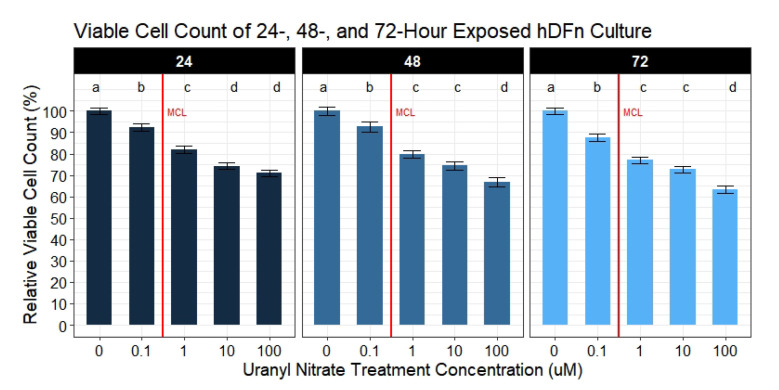
Viable cell count of UN exposed hDFn. Bar charts are faceted by exposure time representing the mean viable cell count percentage ±StdErr of each treatment group (n = 6 done in sextuplicate). All UN concentrations are in μM with -0- representing control group. At all days UN levels below EPA MCL caused a significant decrease in viable cell count. Statistically significant differences between groups are illustrated by compact letter display with different letters at the top representing a balanced Tukey pairwise *t*-tests where *p*-value ≤ 0.05. Group means followed by a common letter were not different at 5% level of significance. Vertical red line illustrates the EPA MCL threshold for uranium (0.126 μM).

Dose-dependent decreases in the viable fibroblast count were measured at 24, 48, and 72 h (Figure 2). Fibroblasts underwent a significant decrease in the viable cell count at concentrations below and above the USEPA MCL. One day of exposure at levels below the MCL significantly decreased the viable cell count by an average of 7.8% (95% CI, 3.7–11.8). Across all levels at 24 h, UN significantly decreased the viable cell count between 7.8% and 29% (95% CI, 3.7–33). Contrary to metabolic activity, after 48 h, no increase in hDFn viability was observed. Levels below the MCL had a similar 7.3% decrease (95% CI, 2.9–11.7) of cells with a range of effects between 7.3% and 33.3% (95% CI, 2.9–37.7) across all concentrations. Notably, hDFn did not undergo a dose-dependent response to levels between 1 μM and 10 μM, with both groups having an averaged 22.9% (95% CI, 18.5–27.3) decrease in the viable cell count. The largest effect on dermal fibroblasts were observed after 72 h of exposure (Figure 2). Levels below the MCL resulted in a significant 12.6% (95% CI, 9.1–16.1) decrease in cell viability, and the highest concentration decreasing the count by 36.7% (95% CI, 33.2–40.1). Similar to 48 h, no dose-dependent decreases of cell membrane integrity were recorded between 1 μM and 10 μM, with an averaged 25.2% (95% CI, 21.7–28.7) decline in viability relative to control. The ED at all time-points were 0.1 μM UN.

### 3.2. Average Metabolic Activity per Well (Metabolic Ratio) Is Increased in Response to UN

**Figure 3:**
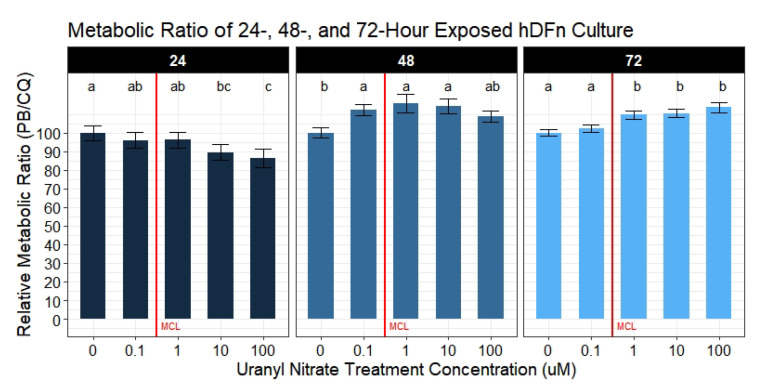
Metabolic ratio of UN exposed hDFn. Bar charts represents the mean relative metabolic ratio percentage ±StdErr of each treatment group (n = 6 done in sextuplicate for each condition). All UN concentrations are in μM with -0- representing control group. Statistically significant differences between groups are illustrated by compact letter display with different letters at the top representing a balanced Tukey pairwise *t*-tests where *p*-value ≤ 0.05. Group means followed by a common letter were not different at 5% level of significance. Vertical red line illustrates the EPA MCL threshold for uranium (0.126 μM).

Individually, the metabolic activity and cell viability are decreased in a dose-dependent manner to UN. However, when calculating metabolic activity normalized to the number of viable cells present, we observe a surprising increase in the metabolic reading per well with increasing UN levels (Figure 3). At 24 h, there is no significant difference in the metabolic ratio of cells across UN levels less than or equal to 1 μM and only produced a 3.8–4.1% (95% CI, 2.7–10.8) change. Cells exposed at the highest concentration resulted in a significant 13.7% (95% CI, 6.6–21.1) averaged decrease in the metabolic ratio. Interestingly, after 48 h, we observed a significant increase in the hDFn metabolic ratio at levels below and above the EPA MCL (Figure 3). Levels below the MCL resulted in a significant 12.5% (95% CI, 5.5–19.5) elevation of metabolic activity in hDFn when normalized to the number of viable fibroblasts. This average increase in the dermal cell metabolic ratio was consistent for 1 μM and 10 μM. Surprisingly, no significant increase in the metabolic ratio was observed from 100 μM at 48 h; nonetheless, there was a recorded 8.7% (95% CI, 1.7–15.7) average increase in the metabolic ratio. Seventy-two hours post-exposure, the metabolic ratio below the MCL was statistically equivalent to the control; however, levels above remained significantly elevated (Figure 3). Just above the MCL, fibroblasts in 1 μM exposure produced an average 9.7% (95% CI, 5.8–13.6) increase in the metabolic ratio. The results from samples challenged with 10 μM were very similar to 1 μM and increased the metabolic ratio by 10.6% (95% CI, 6.7–14.5). Contrary to 24 and 48 h, we recorded the hDFn metabolic ratio in 100 μM to be significantly increased by an average of 13.7% (95% CI, 9.8–17.6).

### 3.3. Growth Curve Population Count Decreased in UN-Treated Groups But Not Proliferation Rate

**Figure 4:**
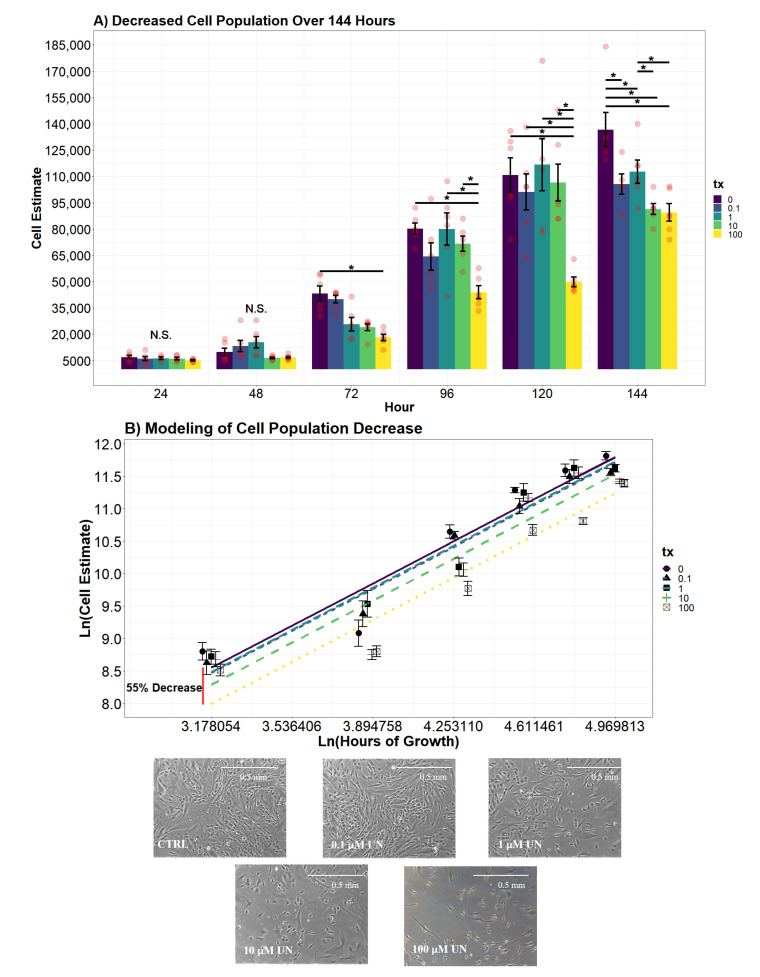
(**A**) Fibroblast cell population reduced when exposed to increasing levels of UN over 144 hour growth curve (n = 6 for each level of UN). Cell counts were identically and independently collected. Contrasts were performed by a Tukey pairwise *t*-test across significant variables from an interaction two-way ANOVA. Significance bars, marked with “*”, refer to significant Tukey pairwise *t*-tests between the conditions at the beginning and the end of the bar. Days where no significant differences were observed are labeled with N.S. (**B**) Modeled regression lines of ln(cell estimate) growth over 144 h (R${}_{adj}^{2}$ = 0.8788). Colors of least squares (LS) lines differentiate UN levels. LS best fit lines represent predicted Ln(cell estimate) based on UN concentration and Ln(hour). Points represent mean cell estimate for each group ±StrErr. Red vertical line represents the largest percent cell estimate difference observed between 100 μM and control. Images were taken using light microscopy and used to illustrate differences in cell population after 72 h of exposure (100× total magnification).

We performed direct cell counts every 24 h across six days and detected diminished populations when challenged with UN, which were exacerbated by higher concentrations (Figure 4A). Cell population estimates did not deviate from the control in the first 48 h of growth. After 72 h of growth, we observed a significant decrease in the cell population between control and 100 μM (*p*-value = 0.0219). On average, 100 μM had approximately 25,000 fewer cells than non-treatment control. On the fourth day of growth, all groups, except 0.1 μM, had significantly more cells than the 100 μM group (*p*-value < 0.001). After 120 h of growth with constant contact of UN, cells in 100 μM had a significantly lower cell population than all other groups (*p*-value < 0.01). Compared to the control, cells exposed to 100 μM for 5 days had, on average, 61,000 fewer cells (95% CI, 4932–84461). All other treatment groups were statistically equivalent to control with an average of 110,000 cells (95% CI, 88,000–120,000). At 144 h of growth, we observed a monotonic decline in the cell population as a result of the UN concentration. The control group had a significantly higher cell population than all other groups with approximately 130,000 cells (*p*-value < 0.05; 95% CI, 114000–146,000). Interestingly, there were no differences in the cell population observed between 0.1 μM and 1 μM groups despite the 10-fold increase in UN concentration (*p*-value = 0.9241). However, cells exposed to 1 μM still had a significantly higher cell population than 100 μM groups (*p*-value = 0.0414).

Figure 4B is a graphical representation of the cell counts with modeled regression lines. From the linear model, we concluded cells exposed to 10–100 μM UN resulted in a significant decrease in the population estimate compared to control after just 24 h and persisted across the six days (*p*-value${}_{0-10}$ = 0.0064; *p*-value${}_{0-100}$ < 0.0001). Cells in lower concentrations were statistically equivalent to control causing only a 6.8%–8.5% (95% CI, −26.7–11.4) difference in the population estimate (*p*-value${}_{0-0.1}$ = 0.3813; *p*-value${}_{0-1}$ = 0.4814). Dermal fibroblasts exposed to 10 μM of UN significantly had 26% (95% CI, 8–45) fewer cells than the control on average across the entire growth curve (*p*-value = 0.0064). The highest concentration tested on cultures, 100 μM, resulted in a significant loss of 55% (95% CI, 37–75) relative to the non-treatment group (*p*-value < 0.001). Although we observe a decrease in cell count at high levels of UN, the average proliferation rate per a one-unit increase in hour (i.e., slope) over the entire growth curve did not deviate from control and is visualized by parallel best fit lines (*p*-value = 0.8645).

### 3.4. Population Doublings Delayed with Increased UN Level

**Figure 5:**
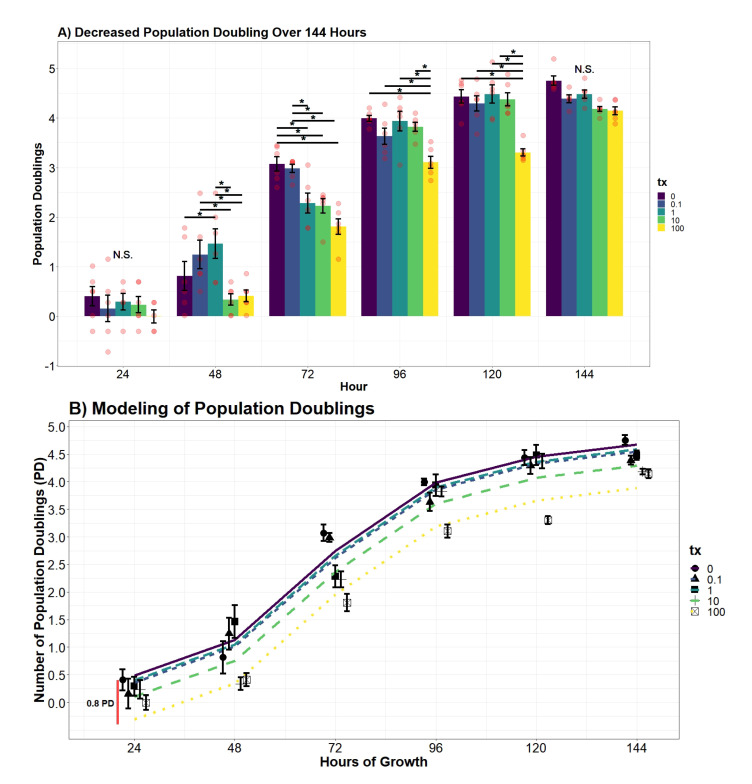
(**A**) Cell population doublings over a 144 h growth curve (n = 6 for each group). Contrasts were performed by a Tukey pairwise *t*-test across significant variables from an interaction two-way ANOVA. Significance bars, marked with “*”, refer to significant Tukey pairwise *t*-tests between the conditions at the beginning and the end of the bar. Time points where no significant differences were observed are labeled with N.S. (**B**) Polynomial regression modeling of hDFn population doublings (R${}_{adj}^{2}$ = 0.9314). Points represent mean PD ±StrErr. Red vertical lines indicates largest PD difference observed between control and 100 μM. Colors of least squares (LS) lines differentiate UN levels.

Utilizing cell counts performed in the growth curve analysis, population doublings (PD) were observed across treatments (Figure 5A). No significant deviations were present within the first 24 h of growth. After 48 h, cells in 1 μM had undergone significantly more PD than control (*p*-value = 0.0435). This group had undergone approximately 1.46 PD (95% CI, −0.5–3.4), whereas the control completed 0.86 PD (95% CI, 0.04–1.59). Interestingly, hDFn in 100 μM had undergone significantly fewer PD in 48 h than all other groups except control and 10 μM (*p*-value < 0.0001). Samples in 100 μM performed only 0.41 PD (95% CI, −1.5–2.3) in 48 h. Fibroblasts in 10 μM, on average, had 0.33 PD (95%, −1.5–2.2) in a 48-hour time span. Control at 72 h had significantly more PD than all other groups, except levels below the MCL. Non-exposed cells and hDFn in 0.1 μM had, on average, 3.1 PD (95% CI, 2.3–3.83) after 72 h of growth. At 96 and 120 h, fibroblasts in 100 μM had significantly less PD than all other groups (*p*-value < 0.001), with fibroblasts exposed to lower concentrations having an average of 4.2 (95% CI, 3.4–5) PD over the two days. No other differences between groups were observed at 96 and 120 h. On the final day of growth, no significant differences were observed across the treatment groups, with each group carrying out an average of 4.8 PD (95% CI, 4–5.5).

Drawing from the 4th-order polynomial model, we concluded that the rate of PD between time points did not vary over the treatment groups; however, the number of PD since inoculation significantly varied depending on the UN concentration (Figure 5B). The average hDFn PD that occurred in 0.1 μM and 1 μM was substantially equivalent to the control over the entire 144-hour growth (*p*-value${}_{0.1}$ = 0.7617, *p*-value${}_{1}$ = 0.9245). Interestingly, cells in 10 μM and 100 μM concentration groups had a significantly lower PD value compared to the control (*p*-value${}_{10}$ = 0.003; *p*-value100 < 0.0001). On average, dermal fibroblasts in 10 μM were 0.38 (95% CI, 0.18–0.59) PD behind the non-treatment group, and the highest UN level setback PD by 0.8 (95% CI, 0.59–1).

### 3.5. In Vitro Percent Closure of hDFn Decreases in the Presence of UN

**Figure 6:**
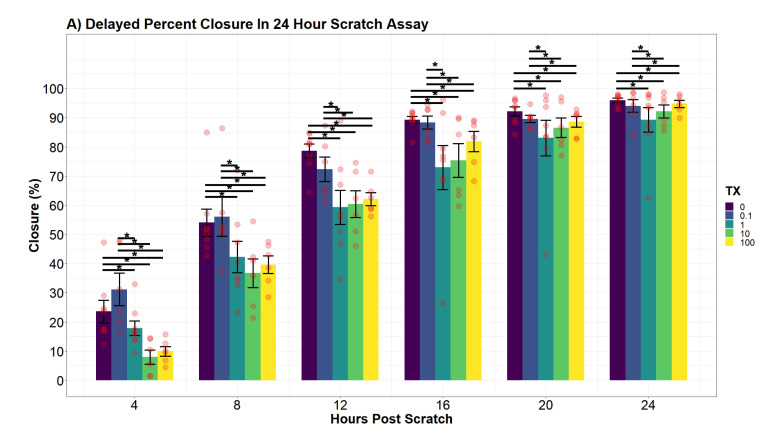

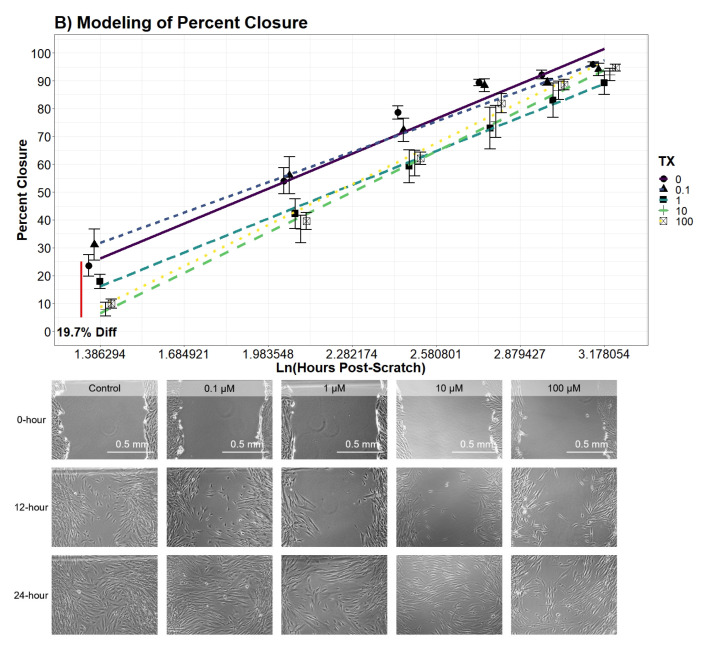
(**A**) Percent closure differences over a 24 h scratch assay (n = 8 for each condition). Contrasts were performed by a Tukey pairwise *t*-test across significant variables from an additive two-way ANOVA. Significance bars, marked with “*”, refer to significant Tukey pairwise *t*-tests between the conditions at the beginning and the end of the bar. (**B**) Interaction modeling of percent closure across treatments (R${}_{adj}^{2}$ = 0.8663). Displayed points represent the mean of each group ±StdErr. Red vertical line represents largest difference observed 4 h post-scratch between control and 10 μM. Inverted light microscopy of hDFn migration at 0, 12, and 24 h post scratch (100× total magnification). All initial scratch widths were 1 mm ± 0.1 mm, with no significant differences of beginning scratch width at hour 0.

From the additive two-way ANOVA, we observed the same pattern of differences across all treatment groups at every time point (Figure 6A). At each hour post-scratch, control cells were significantly more closed than cells in 1, 10, and 100 μM (*p*-value < 0.0001). Additionally, cells exposed to levels below the EPA MCL were observed to insignificantly deviate from control but were significantly more closed than samples in 1, 10, and 100 μM (*p*-value = 0.0001).

Using the interaction regression model, we recorded no deviations in cellular percent closure at levels below the EPA MCL throughout the entire 24-h scratch assay when compared to control (Figure 6B; *p*-value > 0.05). The slope of the closure rate was also found to be statistically equivalent at 0.1 μM and 1 μM compared to control (*p*-value${}_{0.1}$ = 0.1555, *p*-value${}_{1}$ = 0.6976). The control on average increased 3.7% (95% CI, 3.61–3.9) per hour across the entire day. Although fibroblasts in 1 μM had a statistically equivalent slope to control (3.71% h${}^{-1}$; 95% CI, 3.36–3.96), UN still impacted percent closure by significantly reducing the predicted closure percentage by 11% (95% CI, 3.8–18.1) 8 h post-scratch (*p*-value = 0.0003). This significant effect was observed for the remainder of the assay. Although modeling favored an interaction term to reduce variance, hence assigning a unique slope per treatment group, no significant differences in slope coefficients were observed at 10 μM and 100 μM (3.88% h${}^{-1}$; 95% CI, 3.6–4.11; 3.9% h${}^{-1}$; 95% CI, 3.61–4.13; *p*-value${}_{10}$ = 0.08556; *p*-value${}_{100}$ = 0.05985). Nonetheless, similar to hDFn exposed to lower concentrations, 10 μM and 100 μM groups had significantly reduced percent closure 4 h post-scratch by 19.7% (95% CI, 6.5–33) and 17.5% (95% CI, 4.2–30.7). The decrease in cellular percent closure at 100 μM remained significant until 20 h post-scratch, where UN only hindered percent closure by 5.7% (95% CI, −2.64–14.1). Finally, 10 μM exposed groups had a significant reduction in percent closure until 24 h, where we observed only a 7.8% (95% CI, −1.95–17.6) decrease.

## 4. Discussion

Topical exposure to U is an overlooked risk and its impact is not fully understood. In the current study, we sought to describe the chemotoxicity of UN exposure on skin cells and evaluate current levels the EPA deems a safe exposure limit. Thus, cultures of human neonatal dermal fibroblast cells were treated with UN at a range of levels observed in many waters resources in the United States Southwest region. We analyzed the UN biological effects from 24 h up to 144 h to mimic acute contact [20]. Using statistical models to accurately explain the variation in data, we concluded the majority of our hypotheses were supported in predicting UN to negatively impact cell physiology. We also observed UN to induce adverse outcomes to a variety of cell behaviors required to maintain and repair skin. Specifically, we cataloged deleterious outputs in cell membrane integrity, migration measured by percent closure, proliferation, and doubling time when exposed to environmentally relevant levels of UN. At the same time, we observed an interesting effect of UN increasing the average metabolism of hDFn cells when normalized to the number of viable cells present. These changes in cell physiology suggest damage to skin and the exacerbation of pre-existing integument diseases may result if contact with UN-contaminated water occurs.

A handful of published studies reported that U reduced various metabolic processes in leukocytes, kidney, and liver cells [21,30,31,32]. For example, UN exposure has been documented to significantly inhibit phagocytosis in lung macrophages at 50 μM and above [7]. These studies attributed diminished phagocytosis to a reduction in cellular aerobic respiration via measuring the metabolism through popular assays using 3-(4,5-dimethylthiazol-2-yl)-2,5-diphenyltetrazolium bromide or MTT. Other studies on isolated kidney mitochondria presented evidence of UN handicapping succinate dehydrogenase (Complex II) ability to produce ATP through similar assays [33]. However, no study has normalized the metabolic activity to the number of viable cells present. Failure to do this may lead to inappropriate conclusions drawn from many routinely used colorimetric and metabolic-specific assays. Our novel approach in calculating a metabolic ratio using PrestoBlue${}^{\mathrm{TM}}$ and CyQuant Direct${}^{\mathrm{TM}}$ afforded the ability to determine if metabolic disturbances are a result of an actual decrease in metabolism or a byproduct of the cell number. With that said, we collected substantial evidence demonstrating UN to actually increase cellular metabolism at potencies below and above the EPA MCL; however, these outcomes were not seen until 48 and 72 h of exposure. By calculating the ratio of the metabolism to the number of viable cells, we were able to parse out these results. To understand the errors in solely relying on a single assay to evaluate the metabolism, we also analyzed the metabolic activity separately. This type of approach is commonly reported in mammalian and microbial metabolic assays [34,35]. By analyzing metabolism without correcting for the viable cell count, we observe UN to cause a dose-dependent decrease. Although, applying the correction, we rapidly realize that these are erroneous conclusions, and only through the ratio do we obtain an accurate metabolic reading in response to UN. Furthermore, utilizing metabolic assays to assess proliferation poses another flaw. Many studies rely on metabolic assays when drug screening potentially cytotoxic therapeutics; however, these same studies fail to quantify the cell number simultaneously [36,37,38,39]. Thus, opening the potential for inaccuracies in reporting true decreases in the cell number as a response to the drug versus a slowing of metabolism. The latter, as shown in our study, may rebound with increased exposure time. Nonetheless, the increases in metabolic activity observed here are hypothesized to be an outcome of a multitude of internal cell survival mechanisms. We hypothesize these elevated responses in metabolism with increasing time is a byproduct of upregulated apoptosis. Previous studies on other cultured tissues at levels from 100 μM to 1 mM have presented evidence for cytochrome-c and caspase-3/9 dependent necrosis [16,40]. However, these factors are also found to mediate intrinsic apoptosis; therefore, these same mechanisms may be responsible for the observed increase in metabolism at lower levels of UN.

Our second aim was to evaluate if UN induces a dose-dependent decrease in the viable cell count. We observed UN to decrease the viable cell count in hDFn cells at all levels tested and at all time points. Under this circumstance, there is more evidence to suggest the previously mentioned apoptosis hypothesis. Utilizing the semi-permeable membrane, we quantified cells with intact outer membranes and deemed them as viable [21]. As the UN concentration increased, the outer membrane of hDFn cells appear to be proportionally compromised. Using the CyQuant Direct${}^{\mathrm{TM}}$ assay and its DNA binding characteristic, we concluded that UN can present its chemotoxicity after just 24 h at levels below EPA MCL. In the most extreme case, UN decreased the viable cell count by 29% at 100 μM exposure. Surprisingly, concentrations deemed as safe by the USEPA were also found to significantly decrease viability by 7.8% in just 24 h. These results were consistent after 48 and 72 h of exposure. As mentioned previously, we attribute this to be an outcome of UN-induced primary necrosis or intrinsic apoptosis. Cells undergoing necrosis display a fragmented outer membrane and via CyQuant Direct${}^{\mathrm{TM}}$ would be labeled as nonviable [41,42,43,44]. Additionally, non-resolved apoptosis in the absence of phagocytic cells results in secondary necrosis, which displays similar membrane fragmentation characteristics as primary necrosis [45,46]. Therefore, suggesting future experimentation to perform transmission electron microscopy to visualize the deterioration of the outer membrane and caspase-3 assays to confirm these hypothesis. Together, these texts would be a novel finding when using the levels tested here.

Successful wound repair relies on the completion of resident cells’ ability to undergo division and locomotion. If repair of the integument is delayed, it may leave the patient susceptible to opportunistic pathogens and chronic-wound formation [46]. In this study, we examined the influence UN has on the dermal component of phase II wound healing that is characterized by cellular proliferation and migration of both dermal and epidermal cells. The results of the study concluded UN produces considerable deviations in proliferation, cell doublings, and migration in fibroblasts. Fibroblast cells exposed to UN levels greater than or equal to 10 μM displayed significantly delayed proliferation. Strikingly, we observed UN to cause no significant deviations in proliferation rate in either cell counts or calculated population doublings. Instead, our model concluded that there was a significant offset in cell number and PDs over the entire growth curve. It is hypothesized that the delay in mitosis may be attributed to U being able to keep cells locked in S and G2 phases of the cell cycle, as demonstrated in at least one in vivo model [47]. An alternative hypothesis is that the delay in proliferation is once again a product of elevated necrosis or apoptosis. Effectively, reducing the number of viable mitogenic cells present to undergo mitosis. Both avenues for future research are valid based on the data presented here and will be pursued.

In the literature, there is limited information recording percent closure rates of hDFn from UN exposure. An earlier study was published using uranyl acetate and observed a decrease in percent closure; however, this study did not investigate sub-EPA MCL levels with a commonly found species of uranium, UN [48]. Overall, our results drew similar conclusions to Pinto et al. (2016) with large doses of UN, but our current study elucidated percent closure is also significantly hindered at levels below 25 μM. Through modeling and the application of an established scratch assay to measure cell migration, we were able to explain the variation of percent closure more effectively and tease out previously hidden outcomes of UN [29]. Therefore, we concluded levels lower than that previously tested still present deleterious effects on cell locomotion. Similar to the growth curve, no significant differences were found in the percent closure rate amongst the treatment groups, with all treatments closing at approximately 3.74% per hour. Nonetheless, our models presented evidence for a significant average difference in the percent closure across the entire scratch assay. For example, per day, 100 μM treated cells were 27.5% behind the control in percent closure readings. Likewise, cell percent closures while in contact with 10 μM were on average 29% lower than that of control. Surprisingly, percent closures of 0.1 μM and 1 μM were not found to significantly diverge from control and, in some cases, slightly increased closure by 13%. Although not investigated here, the probing of known factors to stimulate fibroblast migration, such as TGF-β1 and -3, matrix metalloproteases (MMP), and FGF-2, should be of focus for future heavy metal research involving fibroblasts and UN [49]. As the outcomes in percent closure may be attributed to variations of expressions of chemotactic factors induced by UN. Other environmental contaminants, such as arsenic trioxide, have already been linked to manipulating migration factors; thus, we hypothesize U may work through similar pathways [49].

The current study has some limitations that should be disclosed for designing future experiments. Here, we subjected in vitro hDFn cultures to acute exposures of UN at levels found in the Southwest United States. Although we observed acute exposure to cause injurious outcomes in cells, we propose subjecting cultured cells chronically to the levels practiced in this study. This would involve exposure to UN over generations with continued contact over multiple passages. By exposing over multiple generations and passages, potential epigenetic changes may manifest into more noticeable physiological changes. Secondly, we investigated only UN; however, U is a co-contaminate in common wells on the Colorado Plateau and is accompanied by arsenic [1,21]. Future studies that target elucidating potential health risks associated with the United States Southwest demographic are recommended to consider other compounds found in surveyed wells and ground water. Nevertheless, the results observed in the current study still apply to the demographic of interest and are also applicable to the Central United States where co-contamination of U and nitrate is the primary concern in aquifers an estimated 1.9 million people use [50,51,52].

As mentioned, the issue addressed here is not only a Southwest United States concern, but is present in the MidWest United States and third world countries where environmental contaminants in water supplies are noticeably higher than predicted [53,54,55]. For example, in war-stricken countries, such as Iraq, Saudi Arabia, and Afghanistan, the mining and military presence have resulted in elevated levels of U in groundwater. In some areas of the Middle East, water has been documented to contain upwards of 1443 μg L${}^{-1}$ U, well above the United States MCL, which is nonetheless in constant use by local residents. The present data provide a physiological understanding on the hazards acute topical exposure may have on communities living in such areas. As noted, particularly on the Navajo Nation, where health disparities are disproportionately higher than the general U.S. population. Uranium’s ability to decrease cell viability, slow proliferation, and prolong migration threatens to exacerbate this health inequality. Furthermore, metal contamination in groundwater is an expanding global health hazard. With this in mind, it is imperative science continues research in understanding the effects metal presence in the environment can have on ecology and most importantly human health.

## 5. Conclusions

Our report shows that UN may have the potential to dramatically impact wound healing via deviation of normal cellular physiology. Dermis cells exposed to UN underwent significant decreases in cellular viability and proliferation. Inconsistent with current literature, our data showed an increase in metabolic activity with increasing concentrations of UN accompanied with prolonged exposure. By taking the ratio of metabolic activity and the number of viable cells, we were able to more accurately differentiate variations in metabolism in response to UN. We further elucidated a substantial decrease in average percent closure at environmentally relevant levels of UN found in the Southwest and Central United States. 

## Data Availability

Data files and R script available for download at: https://github.com/ahrisen?tab=repositories (accessed on: 1 July 2021).
